# Fatal Deterioration of a Respiratory Syncytial Virus Infection in an Infant with Abnormal Muscularization of Intra-Acinar Pulmonary Arteries: Autopsy and Histological Findings

**DOI:** 10.3390/diagnostics14060601

**Published:** 2024-03-12

**Authors:** Nunzio Cosimo Mario Salfi, Gianluca Vergine, Maurizio Poloni, Sara Metalli, Barbara Bigucci, Francesca Facondini, Gianmatteo Pedrazzi, Francesca Masciopinto, Laura Bernabè, Vittorio Sambri, Maria Paola Bonasoni

**Affiliations:** 1 Pathology Unit, Infermi Hospital, Viale Settembrini, 2, 47900 Rimini, Italy; nunziocosimomario.salfi@auslromagna.it; 2 Department of Pediatrics, Infermi Hospital, Viale Settembrini, 2, 47900 Rimini, Italy; gianluca.vergine@auslromagna.it (G.V.); maurizio.poloni@hotmail.it (M.P.); sara.metalli@auslromagna.it (S.M.); barbara.bigucci@auslromagna.it (B.B.); 3 Anesthesia and Intensive Care Unit, Infermi Hospital, Viale Settembrini, 2, 47900 Rimini, Italy; francesca.facondini@auslromagna.it (F.F.); gianmatteo.pedrazzi@auslromagna.it (G.P.); francesca.masciopinto@auslromagna.it (F.M.); laura.bernabe@auslromagna.it (L.B.); 4 Unit of Microbiology, The Greater Romagna Area Hub Laboratory, 47522 Cesena, Italy; vittorio.sambri@unibo.it; 5 Department of Experimental, Diagnostic and Specialty Medicine (DIMES)—Alma Mater Studiorum, University of Bologna, 40138 Bologna, Italy; 6 Pathology Unit, Azienda USL-IRCCS di Reggio Emilia, Via Amendola 2, 42122 Reggio Emilia, Italy

**Keywords:** respiratory syncytial virus, bronchiolitis, anomalous pulmonary lobation, abnormal muscularization of intra-acinar pulmonary arteries, sudden death

## Abstract

Respiratory syncytial virus (RSV) infection represents a global and noteworthy cause of hospitalization and death in infants of less than 1 year of age. The typical clinical manifestation is bronchiolitis, an inflammatory process of the small airways. The symptoms are usually a brief period of low-grade fever, cough, coryza, breathing difficulties, and reduced feeding. The progression of the disease is difficult to predict, even in previous healthy subjects. Symptoms may also be subtle and underestimated, thus leading to sudden unexpected infant death (SUID). In these cases, RSV infection is discovered at autopsy, either histologically or through real-time reverse transcription polymerase chain reaction (RT-PCR) performed on nasopharyngeal swabs. Herein, we describe a case of RSV infection in a 6-month-old infant with no risk factors, who rapidly deteriorated and unexpectedly died of respiratory insufficiency in a hospital setting. RT-PCR on nasopharyngeal swabs revealed RSV. The autopsy showed diffuse lymphogranulocytic bronchitis and bronchiolitis, and multiple foci of acute pneumonia. Abnormal muscularization of the intra-acinar pulmonary arteries was also observed, which likely contributed to worsening the lung impairment.

## 1. Introduction

Respiratory syncytial virus (RSV) infection represents a global and noteworthy cause of hospitalization and death in infants less than 1 year of age [1]. 

By the time children reach one year of age, approximately 60–70% have encountered RSV infection (with 2–3% of those cases resulting in hospitalization), and nearly all children have experienced RSV infection by the age of two [2]. Roughly, 25% to 40% of infants and children exhibit signs or symptoms of bronchiolitis or pneumonia upon their initial exposure to RSV [3].

RSV presents seasonal outbreaks depending on the geographical hemisphere. In the northern hemisphere, infection spreading ranges from November to April. In southern United States (US), comprising Florida, the RSV season is longer, usually starting in July. In tropical areas like Puerto Rico in the US, as well as in equatorial regions such as the Philippines and Mozambique, the RSV season may persist throughout the year with minimal fluctuations [4]. In infants less than 1 year of age, the typical clinical manifestation of RSV infection is bronchiolitis, an inflammatory process of the small airways. The symptoms are usually a brief period of low-grade fever, cough, coryza, breathing difficulties, and reduced feeding. The illness can exacerbate and peak between day 3 and 5 since onset. Infants less than 6 weeks of age must be carefully observed as apnea can be the only clinical sign [5]. 

RSV infection can be severe with higher morbidity and mortality in infants with the following risk factors: prematurity, neuromuscular diseases, chronic lung disease of prematurity or bronchopulmonary dysplasia, congenital cardiovascular malformations, abnormalities of the airways, and deficiencies of the immune system [6]. RSV in infants with Down syndrome, cystic fibrosis, or cerebral palsy usually require hospitalization. However, 85% of infants with RSV bronchiolitis admitted to pediatric wards do not present significant risk factors [7]. Moreover, the progression of the disease is difficult to predict, even in previously healthy subjects [8]. Symptoms may also be subtle and underestimated, thus leading to sudden unexpected infant death (SUID). In these cases, RSV infection is discovered at autopsy, either histologically or through real-time reverse transcription polymerase chain reaction (RT-PCR) performed on nasopharyngeal swabs [9,10]. 

Herein, we describe a case of RSV infection in a 6-month-old infant with no risk factors, who rapidly deteriorated and unexpectedly died of respiratory insufficiency in a hospital setting. RT-PCR on nasopharyngeal swabs revealed RSV. The autopsy showed diffuse lymphogranulocytic bronchitis and bronchiolitis, and multiple foci of acute pneumonia. Abnormal muscularization of the intra-acinar pulmonary arteries was also observed, which likely contributed to worsening the lung impairment. 

## 2. Case Report

### 2.1. Clinical Evolution

**Figure 1:**
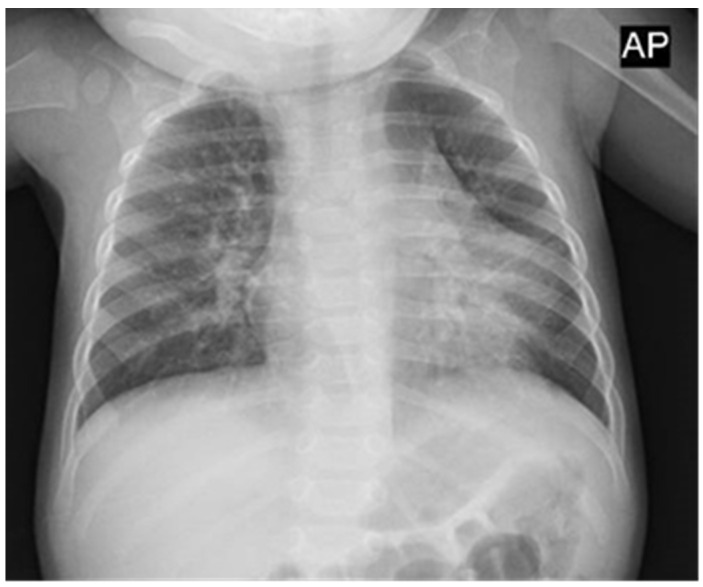
Chest X-rays: diffuse bilateral peribronchial thickening in the hilar and perihilar regions with alveolar confluence in the left perihilar inferior area. AP: anteroposterior.

The patient was a 6-month-old female infant born at term, previously healthy, with no known pathologies. She was admitted to the Emergency Department because of fever (38.7 °C) and cough, associated with drowsiness, decreased feeding, and two episodes of vomit. At physical examination, she presented pharyngeal hyperemia, and chest auscultation identified a slightly harsh vesicular murmur; however, she presented overall good general conditions, with a respiratory rate of 29 breaths/min, a cardiac frequency of 190 beats/min, and arterial oxygen saturation (SaO_2_) of 97%. Chest X-rays showed diffuse bilateral peribronchial thickening in the hilar and perihilar regions, with alveolar confluence in the left perihilar inferior area (Figure 1).

Two hours after admission, the infant presented an episode of vomit, but she remained in a good state. The laboratory test results were as follows: a white blood cell count of 11.86 × 10^9^/L, a red blood cell count of 4.08 million/mm^3^, hemoglobin levels of 10 g/dL, C-reactive protein levels of 9 mg/L (the normal value is less than five), platelets at 535 × 10^3^/mcL, sodium at 134 mEq/L, and potassium at 4.7 mEq/L. Given the presence of alveolar confluence on the X-ray and considering the age, it was decided to start a treatment with intravenous ceftriaxone. 

Three hours after admission, the infant suddenly became dyspneic, cyanotic, and hyporeactive, with deviation of the eyes. Flow oxygen therapy was promptly given. At chest auscultation, the vesicular murmur was overall reduced. The heartbeat was 95/min and the SaO_2_ was 80%. Pulse rate was detected with decreased width. Oxygen inflation was shifted to positive pressure ventilation through a bag valve mask. She had a cardiopulmonary arrest (CPA) and the initiation of external cardiac massage allowed for the recovery of the pulse and breathing after one minute. Endotracheal intubation was performed, and the patient presented a second CPA. Intravenous adrenalin was started at 0.1 mg/3–4 min and two boluses of 60 mL crystalloids were administered. Venous blood gas analysis revealed a pH of 6.99, PCO_2_ at 75 mmHg, HCO_3_^−^ at 23 mmol/L, a base excess of −13, and lactate at 11.9 mmol/L. The electrolytes were within normal range. Electrocardiogram monitoring displayed pulseless electrical activity (PEA) and cardiopulmonary resuscitation (CPR) was continued. Echocardioscopy was performed and no cardiac anomalies were observed. Due to PEA persistence, 8.4% 6 mEq sodium bicarbonate was injected. Venous blood gas analysis showed worsened values, with a pH of 6.76, PCO_2_ at 78 mmHg, HCO_3_^−^ at 40 mmol/L, a base excess of −24, lactate at 18.99 mmol/L, and potassium at 4.7 mEq/L. After two hours of continuous CPR, and five hours after hospital admission, the infant was pronounced dead. 

Subsequently, a nasopharyngeal swab for respiratory viruses was executed and RSV was isolated by RT-PCR. IL-6 testing was requested on previous stored blood and the result was 22.1 pg/mL (normal range: <5.9). 

### 2.2. Autopsy Findings 

A post-mortem examination was carried out to comprehend the rapid exacerbation of the clinical course and to discover the cause of death. 

At autopsy, the infant appeared well-nourished, appropriately grown for age, with no external dysmorphisms. The body weight was 5140 g and the anthropometric measurements were as follows: a crown–heel length of 60 cm, a crown–rump length of 41 cm, a head circumference of 43 cm, a chest circumference of 39 cm, an abdominal circumference of 38 cm, an inner canthal distance of 2.8 cm, an outer canthal distance of 7.8 cm, a bilateral hand length of 7 cm, and a bilateral foot length of 8 cm. 

**Figure 2:**
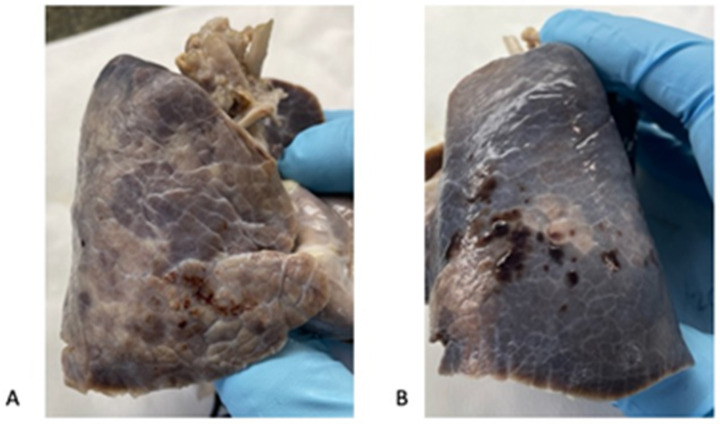
Abnormal lobation of the lungs (formalin-fixed specimens): the right lung (**A**) showed two lobes with an incomplete horizontal fissure. The left lung (**B**) presented one lobe with an almost imperceptible oblique fissure. The lymphatic pattern was marked with irregular areas of inflation. Abundant petechial hemorrhages were seen on the left lung surface (**B**).

**Figure 3:**
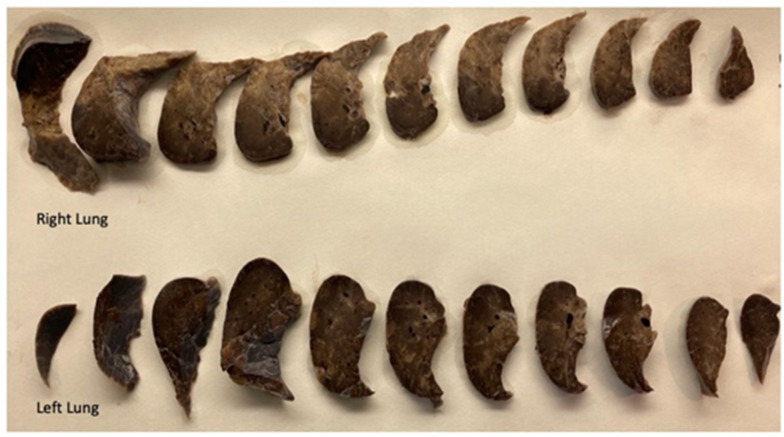
Serial sections of both lungs (formalin-fixed specimens): the parenchymal surface was irregularly mottled with compacted areas and nonuniform areas of air expansion. Note the absence of lobation with indistinct fissures.

The skin was overall pale and irregularly mottled. The internal examination revealed minimal bilateral pleural and pericardial serous effusions of 1 mL each. In the lungs, lobation was abnormal, with two lobes in the right and one lobe in the left, due to incomplete horizontal and almost imperceptible oblique fissures, respectively (Figure 2). Both lungs presented, on the pleural surface, marked lymphatic patterns and nonuniform patterns of inflation. The left lung also showed multiple serosal petechial hemorrhages in the lower lobe. After formalin fixation, both lungs were serially sectioned along the vertical plane. At slicing, they displayed a mottled appearance of the parenchyma, with more compacted regions and irregular air expansion (Figure 3). The lung weight was 74.3 g for the right and 65.6 g for the left, respectively. 

The heart (weighing 35 g) was anatomically normal, with atrioventricular and ventriculoarterial concordance, and regular systemic and pulmonary venous return. The right ventricle presented minimal dilatation. 

**Figure 4:**
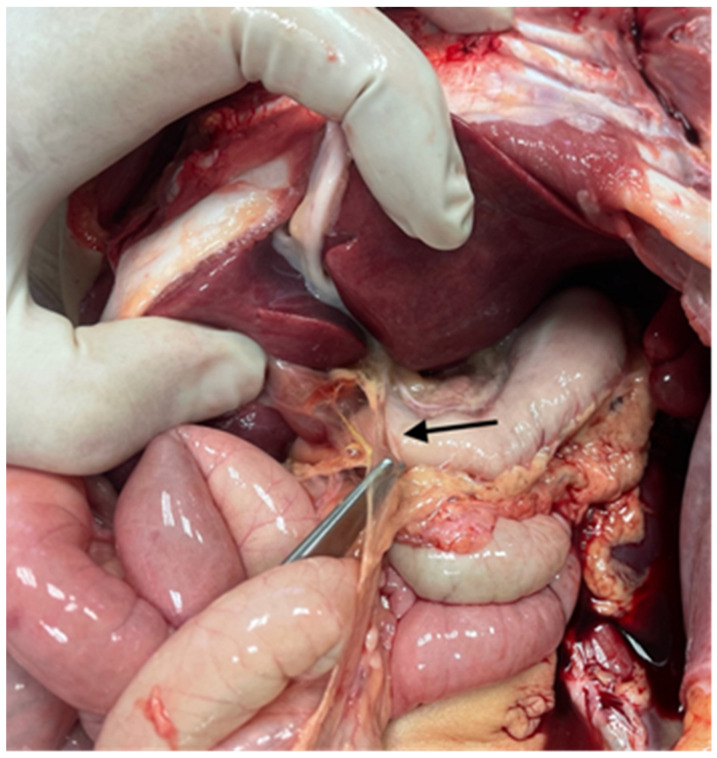
Ladd’s band: the peritoneal band connected the gallbladder, the duodenum, and the transverse bowel (arrow).

**Figure 5:**
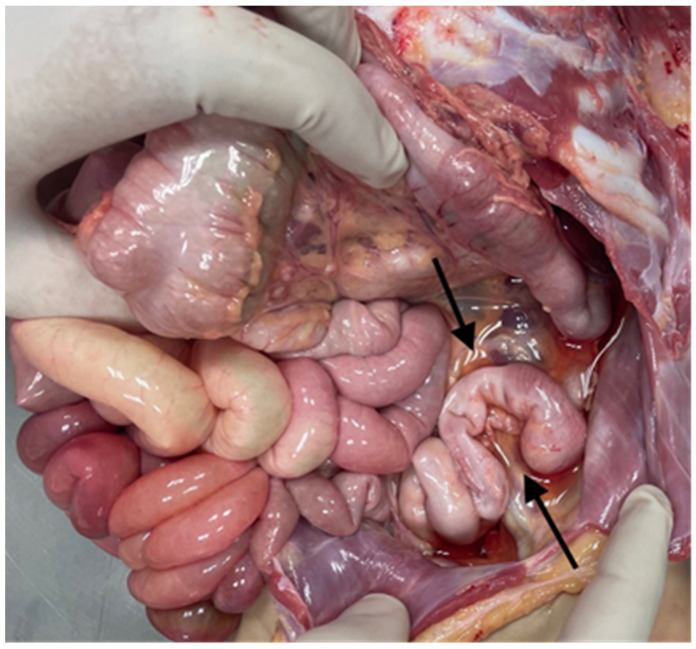
Dolichosigma: the sigma was convoluted (arrows), despite normal intestinal rotation.

The examination of the abdominal organs showed the Ladd’s band between the gallbladder, duodenum, and transverse bowel (Figure 4). Dolichosigma was also detected, but intestinal rotation was normal (Figure 5). 

No other internal anomalies were found. The brain was not examined for body preservation as the parents wanted to see their baby before the funeral.

### 2.3. Histological Findings

**Figure 6:**
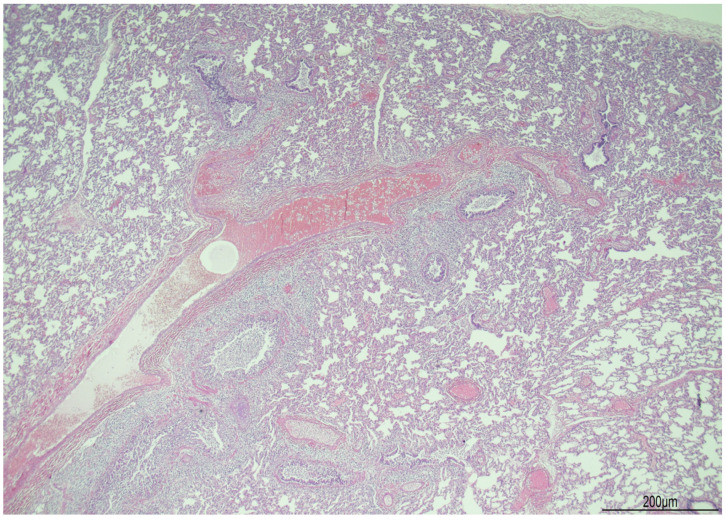
Lung microscopy: diffuse lymphogranulocytic bronchitis and bronchiolitis with extension of the inflammatory infiltrate in the peribronchial and peribronchiolar areas (hematoxylin and eosin 2 HPF).

**Figure 7:**
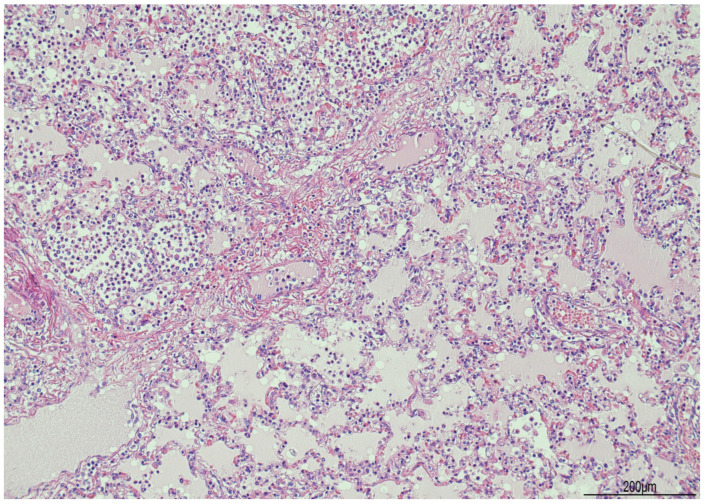
Lung microscopy: acute pneumonia with neutrophils (left side of the picture) and pulmonary edema (right side) were patchy at histological examination when observed (hematoxylin and eosin 10 HPF).

**Figure 8:**
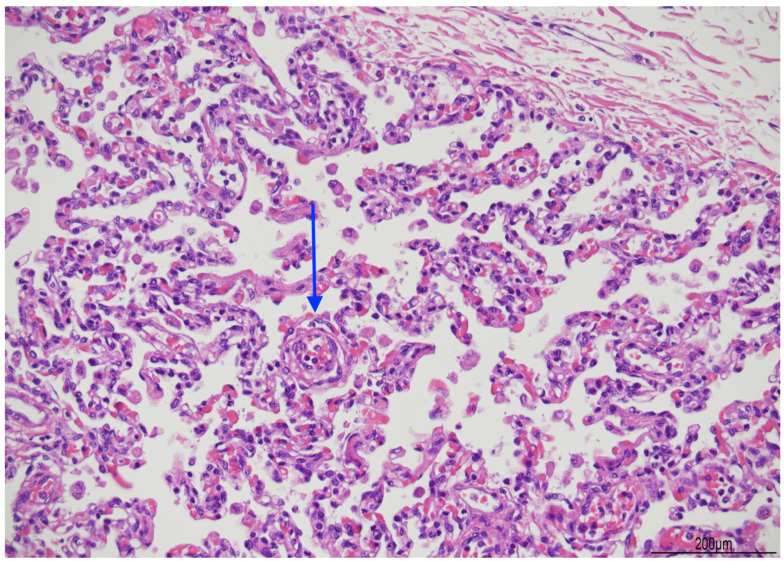
Abnormal muscularization of the intra-acinar arteries: small arteries still maintained the muscular wall (arrow, hematoxylin and eosin 20 HPF).

**Figure 9:**
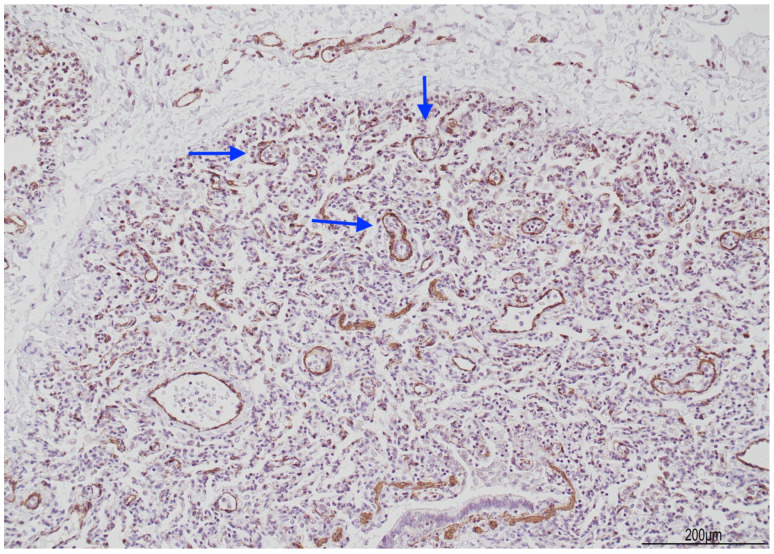
Immunohistochemistry for smooth muscle actin, which highlighted the muscular wall in the intra-acinar arterioles (arrows, 10 HPF).

At histology, the lungs displayed diffuse and severe lymphogranulocytic bronchitis and bronchiolitis with luminal plugging and extension of the inflammatory infiltrate in the peribronchial and peribronchiolar areas (Figure 6). Scattered foci of acute pneumonia with neutrophils and pulmonary edema were also observed (Figure 7). In most of the fields, intra-acinar arteries presented abnormal muscularization, evidenced by immunohistochemistry for smooth muscle actin (SMA) (Figure 8 and Figure 9). 

In the heart, there was very mild and focal lymphocytic myocarditis without necrosis. Few wavy fibers were also observed. The lymphoid system was, overall, activated with hyperplastic lymphoid follicles. 

## 3. Discussion

RSV infection is a recognized cause of SUID, especially in infants less than 6 months of age. The symptoms may be subtle or worsen so rapidly that the diagnosis is made only at autopsy, following evidence of airway inflammation and the isolation of RSV by RT-PCR [9,10]. Bronchiolitis is the main clinicopathological feature of RSV. Distal bronchioles are plugged with mucus, inflammatory infiltrate, and sloughed epithelial cells. This condition easily trap air in the lungs, favouring apneic episodes and sudden death [11]. Many mechanisms have been suggested to explain such a fatal outcome in case of respiratory viral infection. These include the triggering of laryngeal chemoreceptors and the alteration of peripheral chemoreceptors, like the neuroepithelial bodies, specialised in detecting hypoxia and breath control. Therefore, the combination of airway impairment, caused by viral infection, and hypoxia can represent a lethal combination [11]. Premonitory symptoms may be subtle or even absent, despite widespread infection and inflammation found at post-mortem examination [10]. Even in a hospital setting, the illness can rapidly exacerbate, especially in young infants [12,13,14]. Another proposed mechanism of unexpected deaths is a cytokine storm that can trigger acute encephalopathy with multiple organ failure, as proposed by Kakimoto et al. for two patients where a dramatic elevation of IL-6 correlated with sudden death [12]. In our patient, the plasma level of IL-6 was not so high, at 22.1 pg/mL (normal range: <5.9), and there was no evidence of organ failure.

In the case we presented, the infant deteriorated in two hours, after presenting dyspnea, cyanosis, and hypotonia. SaO_2_ fell from 97%, at admission, to 80% before the first CPA. At autopsy, grossly, the lungs showed abnormal lobation: the right lung had two lobes with incomplete horizontal fissures and the left lung was almost unilobed with an indistinct oblique fissure. Abnormal lobation is not uncommon and represents an anatomical variation. However, complete fissures are important during breathing, as they act as a plane of cleavage. During inspiration, the superior part of the lung expands anterolaterally; meanwhile, the inferior part enlarges posteriorly. Incomplete fissures are responsible for collateral ventilation, wherein the alveoli manage air flow disconnected from the main airways, but common to two lobes [15]. In thoracic surgery, incomplete fissures may compromise the patient’s outcome, especially after a lobectomy, because they can determine air leak and prolonged chest drainage [16]. Moreover, incomplete fissures can favour the widespread dissemination of infective diseases, as they are no more confined within a lobe [17]. In the case we described, postmortem histology displayed, in both lungs, diffuse lymphogranulocytic bronchitis and bronchiolitis, with extension of the inflammatory infiltrate in the peribronchial and peribronchiolar areas, also associated with patchy areas of acute pneumonia. Therefore, ventilation impairment was extensive.

In addition, abnormal muscularization of the intra-acinar arteries was observed in most fields, as, at 6 months of age, they should be devoid of smooth muscle in the wall. This is the histologic hallmark of persistent pulmonary hypertension of newborn (PPHN). Newborns with this condition usually manifest respiratory distress and severe hypoxemia at birth or shortly thereafter, because pulmonary artery pressure is increased with right-to-left shunt. Additional oxygenation and mechanical ventilation are needed to reduce adverse and fatal outcomes [18]. PPHN can be associated with many cardiovascular malformations, with an incidence of 0.4–6.8/1000 live births and 5.4/1000 live births in late preterm infants. The mortality rate varies from 7.6% in all newborns to 10.7 in case of severe PPHN [19]. Long-term morbidities have been reported, such as neurodevelopmental sequelae in 25% of infants at 2 years of age [20]. 

In fetal life, oxygenation relies on placental circulation, and pulmonary vascular resistance (PVR) remains high, in order to maintain low pulmonary blood flow (“Qp”). At birth, the oxygenation of tissue depends on pulmonary gas exchange, and the beginning of ventilation is guaranteed through a rapid decrease in PVR, leading to an 8- to 10-fold increase in “Qp”. Failure in this process causes the persistence of high PVR, subsequent severe hypoxemia, and pulmonary hypertension [21]. PPHN has been reported as idiopathic in 10–20% of cases and secondary from abnormal constricted vasculature such as infections (30%), meconium aspiration syndrome (24%), respiratory distress syndrome (7%), and congenital heart disease (6%) [19].

Abnormal muscularization of the intra-acinar arteries has been reported in SUID and interpreted as idiopathic PPHN [22]. This condition may be due to a prolonged intra-uterine hypoxia with the induction of chronic vasoconstriction of small arteries [23,24]. In addition, the persistence of fetal pulmonary circulation can be worsened postnatally by abnormal arterial vasoconstriction, caused by the secondary conditions already mentioned, especially infections [23]. However, smooth muscle proliferation in small pulmonary arteries, due to a hypoxic state, needs several days to fully develop [25]. 

The case we described can be assimilated to a SUID, as the respiratory symptoms fatally deteriorated in almost two hours. Autopsy and histological findings allowed us to understand or at least to hypothesise the pathophysiology of death. As the infant had presented catarrhal cough for one week before hospital admission, RSV infection might have been likely contracted in that period, then bronchitis and bronchiolitis easily extended, probably allowed by the congenital abnormal lobation of the lungs. Abnormal muscularization of the intra-acinar arteries might have been a pre-existent prenatal condition, and vasoconstriction, due to infection and hypoxia, could have further exacerbated the respiratory impairment. On the other hand, it is difficult to assess if the smooth muscle in the arteriolar wall could have been a consequence of airway compromission and hypoxia. However, one week since the onset of infection seemed too short a period. Hypoxia induced vasoconstriction through an irreversible loop leading to death.

## 4. Conclusions

The autopsy and histological examination were fundamental in explaining the death in this specific case. This is fundamental for parents’ bereavement and future counselling. Moreover, careful pulmonary investigations should be carried out in all cases of SUID wherein respiratory viruses are detected, whether associated or not with inflammatory findings, especially in a forensic setting. 

## Data Availability

The data presented in this study are available on request from the corresponding author.
